# Effect of Sensor Set Size on Polymer Electrolyte Membrane Fuel Cell Fault Diagnosis

**DOI:** 10.3390/s18092777

**Published:** 2018-08-23

**Authors:** Lei Mao, Lisa Jackson

**Affiliations:** 1 School of Engineering Science, University of Science and Technology of China, Hefei 230027, China; 2 Department of Aeronautical and Automotive Engineering, Loughborough University, Loughborough LE11 3TU, UK; l.m.jackson@lboro.ac.uk

**Keywords:** PEM fuel cell, sensor selection, fault diagnosis, sensor set size effect, wavelet packet transform

## Abstract

This paper presents a comparative study on the performance of different sizes of sensor sets on polymer electrolyte membrane (PEM) fuel cell fault diagnosis. The effectiveness of three sizes of sensor sets, including fuel cell voltage only, all the available sensors, and selected optimal sensors in detecting and isolating fuel cell faults (e.g., cell flooding and membrane dehydration) are investigated using the test data from a PEM fuel cell system. Wavelet packet transform and kernel principal component analysis are employed to reduce the dimensions of the dataset and extract features for state classification. Results demonstrate that the selected optimal sensors can provide the best diagnostic performance, where different fuel cell faults can be detected and isolated with good quality.

## 1. Introduction

Due to characteristics such as zero emission and high efficiency, hydrogen fuel cells—especially polymer electrolyte membrane (PEM) fuel cells—have attracted much attention in the last few decades. This has led to its widespread use, such as in stationary power stations, consumer devices, and automotives.

However, the reliability and durability of PEM fuel cells are still two major barriers to its further commercialization. To address these issues, a set of studies have been devoted to fuel cell fault diagnosis, from which the fuel cell faults can be detected and isolated—thus, mitigation strategies can be taken to extend fuel cell performance. In these studies, various information can be collected from fuel cell systems and used for fault diagnosis [1,2]. Based on information used in the analysis, these studies can be loosely divided into two categories, including techniques using fuel cell voltage only [3,4,5,6], and methodologies using multiple sensor data [7,8,9,10,11,12,13,14,15,16,17].

As fuel cell voltage change can directly indicate fuel cell performance variation, several studies have employed fuel cell voltage to identify faults in the PEM fuel cell [3,4,5,6]. Yousfi-Steiner et al. [3] investigated the effect of water management strategies on fuel cell voltage degradation. Steiner et al. [4] applied wavelet transform to fuel cell voltages for identifying PEM fuel cell faults, and results demonstrate that fuel cell flooding can be distinguished from the normal state. Kim et al. [5] used the hamming neural network and fuel cell voltage to configure the parameters of an equivalent circuit model, from which the fuel cell faults can be detected and isolated. Li et al. [6] employed individual fuel cell voltages from a PEM fuel cell stack to isolate different faults, including faults from electric circuits, temperature subsystems, and hydrogen and air supply systems.

Compared to the fault diagnosis using fuel cell voltage, more studies have been performed using multiple sensors information [7,8,9,10,11,12,13,14,15,16,17,18,19,20,21]. Both single [7] and multiple PEM fuel cell faults [8,9,10,11,12,13,14,15,16] can be identified accurately with information from multiple sensors, using either model-based or data-driven approaches. Furthermore, in order to increase computational efficiency when dealing with data from multiple sensors, dimension reduction techniques such as principal component analysis have been selected to reduce the dimension of the original dataset, while retaining useful information [17,18,19,20].

It can be found from these studies that no guideline has been provided about the selection of sensor information for reliable PEM fuel cell fault diagnosis, and each of these two kinds of methodologies can be used in certain scenarios to detect and isolate PEM fuel cell faults. Therefore, it is necessary to perform a comparative study on the performance of PEM fuel cell fault diagnosis using information from different sensors, from which the effectiveness of different sensor sets in PEM fault diagnosis can be better clarified.

In this study, a comparative study is presented using information from different sensor sets, including fuel cell voltage sensor only, all the available sensors, and selected optimal sensors, which is defined as the sensors being more sensitive to fuel cell performance changes, and thus being able to provide timely and reliable fuel cell fault diagnostic results. The methodology of selecting optimal sensors will be described in Section 2.3. By comparing their diagnostic performance on isolating the same PEM fuel cell faults, the effectiveness of different sensor sets on PEM fuel cell fault diagnosis can be better clarified. In Section 2, fault diagnostic techniques used in the current analysis are described, including wavelet packet transform and kernel principal component analysis. Section 3 presents a comparative study on the PEM fuel cell fault diagnosis using information from different sensor sets, including fuel cell voltage only, all the available sensors, and selected optimal sensors. Two different PEM fuel cell faults are used for the comparative study, including fuel cell flooding and membrane dehydration. From the findings, conclusions are given in Section 4.

## 2. Fault Diagnostic and Sensor Selection Techniques

### 2.1. Wavelet Packet Transform

As information from different sensor sets are used herein for PEM fuel cell fault diagnosis, several techniques have been selected in the current study to perform fault diagnosis. For fault diagnosis using fuel cell voltage only, wavelet packet transform, which has been widely used in several applications [21,22,23,24], was selected to extract the wavelet coefficient from the fuel cell voltage, expressed in Equation (1). It should be noted that compared to the wavelet transform, wavelet packet transform can extract coefficients from both approximation and detailed terms at each level, thus providing more information from the original data.

(1) $$X_{w}\left(a,b\right)=\frac{1}{{\left|a\right|}^{1/2}}\int_{-\infty }^{\infty }x\left(t\right)\bar{\psi }\left(\frac{t-b}{a}\right)\mathrm{dt}$$

where x(t) is the fuel cell voltage, and ψ(t) is the mother wavelet. Where Morse wavelet is used in the current study, written as Equation (2), a and b are scale and shift parameters, respectively.

(2) $$\psi \left(t\right)=U\left(t\right)a_{\beta ,\gamma }t^{\beta }e^{-t\gamma }$$

where $a_{\beta ,\gamma }$ is the normalizing constant, U(t) is the unit step, β is the decay parameter, and γ characterizes the symmetry of Morse wavelet.

With extracted wavelet coefficients at each level, the normalized energy can be calculated using Equation (3):

(3) $$E^{p}=\frac{1}{N_{p}}\underset{j.k}{\sum }{\left|X_{w}\left(a,b\right)\right|}^{2}$$

where $E^{p}$ is the normalized energy for specific wavelet packet p, $N_{p}$ is the number of coefficients in wavelet packet p.

From generated normalized energies at different levels, the two highest energies were selected and used for fuel cell state classification, as they contained more information on the original signal.

### 2.2. Kernel Principal Component Analysis

With the use of multiple sensor information in the fuel cell analysis, dimension reduction techniques should be used to reduce computational complexity and improve computational efficiency [25,26,27,28,29].

Kernel principal component analysis (KPCA) was selected herein for dimension reduction purposes, since, compared with principal component analysis (PCA), KPCA is more suitable for complex and non-linear systems [29].

The general idea of KPCA is a non-linear mapping of the original data to a higher-dimension space (where they vary linearly), then PCA is applied in the new space. Therefore, the key in KPCA is generating a kernel matrix and then projecting it towards the new direction. Equation (4) expresses the generation of kernel function:

(4) $$K_{i,j\;}=\kappa \left(x_{i},x_{j}\right)$$

where κ is the kernel function, $\kappa \left(x_{i},x_{j}\right)=\phi {\left(x_{i}\right)}^{T}\phi \left(x_{j}\right)$, ϕ creates linearly independent variables from the original data, $x_{i}$ and $x_{j}$.

The generated kernel function is then modified using Equation (5):

(5) $$\tilde{K}=K-1_{n}K-K1_{n}+1_{n}K1_{n}$$

where $1_{n}$ is the n×n matrix where all elements take the value of 1/n.

From Equation (5), the highest L eigenvalues and corresponding eigenvectors ($a_{1}$, $a_{2}$, …, $a_{L}$) can be calculated and projected to the new direction, which is written in Equation (6):

(6) $$z_{l}=\overset{n}{\underset{i=1}{\sum }}a_{\ln}\kappa \left(x_{i},x\right)$$

where $z_{l}$ is the lth element of the projected vector (l∈1, 2,…,L), $a_{\ln}$ is the corresponding value in the above calculated eigenvectors.

The proper number of principal components can be decided using Equation (7):

(7) $$\frac{\sum_{i=1}^{L}\lambda_{i}}{\sum_{i=1}^{n}\lambda_{i}}<T$$

where $\lambda_{i}$ is the ith principal component, n is the number of total principal component, L is the selected number of principal components (with the selected principal components, useful information will not be lost), and T is the threshold value (0.95 was selected in this case, based on previous studies [11,30,31,32]).

### 2.3. Optimal Sensor Selection Methodology

In this study, the performance of selected optimal sensors in detecting and isolating PEM fuel cell faults was investigated and compared to those from other sensor sets. The selection was based on the sensitivity analysis, where sensor sensitivities to variations in fuel cell parameters (representing different failure modes) were calculated with the developed fuel cell model, from which the sensors could be ranked to evaluate their contributions to the fuel cell fault diagnosis [31,32].

The sensor resistance to noise was also considered in the selection, as environment/measurement noise can affect the sensor measurements—especially those insensitive to changes in fuel cell performance. This can be written as the following equation:

(8) $$\left\{\delta P\right\}={\left(S^{T}S\right)}^{-1}S^{T}\left\{\delta R\right\}=G\left\{\delta R\right\}$$

where S is the sensitivity matrix, {δR} is the variation in sensor measurements (due to the noise influence), {δP} is the change in the fuel cell model parameters (representing different failure modes), and G is the gain matrix.

With Equation (8), measurement noise could be simulated, and its influence on the fuel cell fault diagnostic results was then investigated using statistical analysis. From the results of the sensor sensitivities and their noise-resistive abilities, the sensors providing the best diagnostic performance using affordable computational time could be determined. More details of the selection process can be found in the previous study [29].

## 3. Effectiveness of Various Sensor Sets in Polymer Electrolyte Membrane Fuel Cell Fault Diagnosis

### 3.1. Description of Polymer Electrolyte Membrane Fuel Cell Test Bench and Data

**Figure 1:**
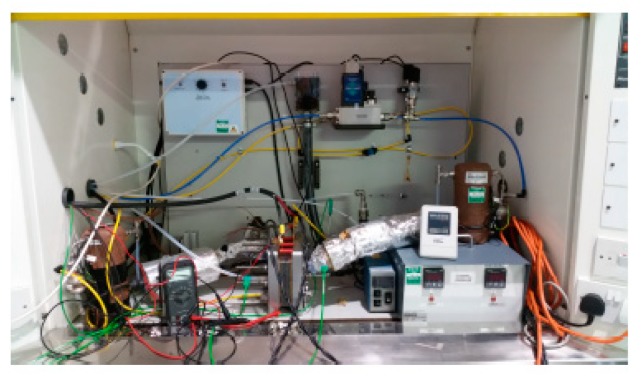
Polymer electrolyte membrane (PEM) fuel cell test bench.

In the current study, an 80 W PEM fuel cell system was used, which included a fuel cell stack, cooling systems, and hydrogen and air supply systems, depicted in Figure 1.

The tested PEM fuel cell had a 100 cm^2^ active area, and was manufactured by Pragma Industries, Biarritz, France using the same materials and technologies as commercial PEM fuel cells, including a Nafion polymer electrolyte membrane, a platinum nano-particle catalyst, carbon diffusion materials, silicone-sealing gaskets, and graphite flow field plates. Table 1 lists the technical details of the PEM fuel cell system.

In order to test the effect of sensor set size on the fault diagnosis performance, a set of sensors, including voltage sensor, thermocouple, flow meter, pressure gauge, and humidification sensor, were installed at different locations of the PEM fuel cell system (anode and cathode outlets) to collect information during the fuel cell operation, listed in Table 2. It should be mentioned that regarding the gas leaving the Nafion tube, bubbler-type humidifiers will pass through a customer-manufactured humidification sensor T-piece, where the humidity levels in the gas stream before entering the fuel cell stack can be measured.

**Figure 2:**
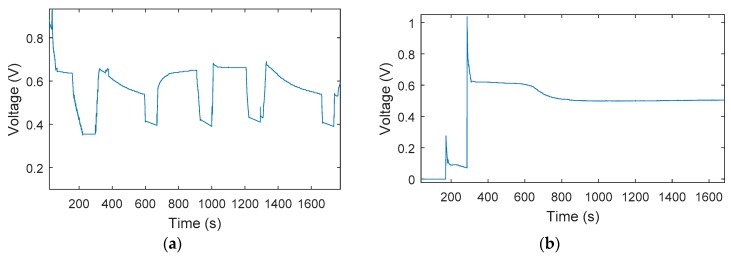
PEM fuel cell voltage drop due to cell flooding and membrane dehydration. (**a**) Cell flooding; (**b**) membrane dehydration.

**Figure 3:**
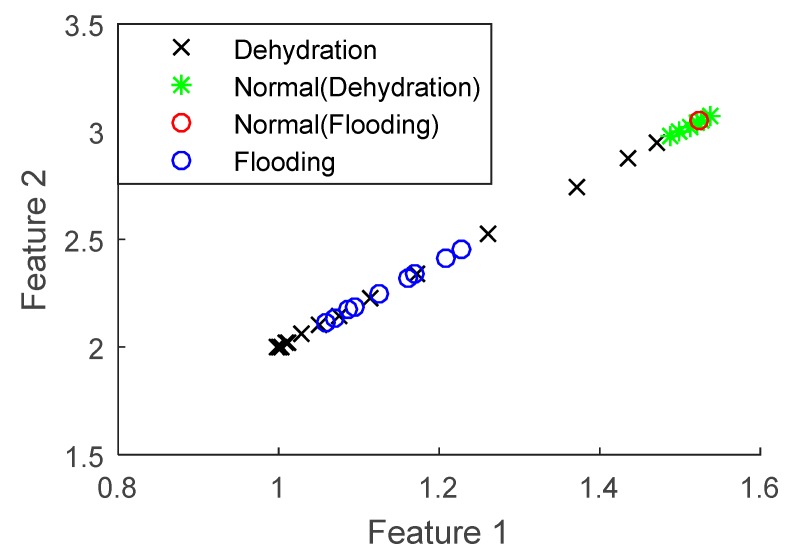
Fault diagnosis performance using voltage only.

Two different PEM failure modes, including fuel cell flooding and membrane dehydration, were tested with the above-mentioned test bench. To achieve the flooding scenario, the liquid water inside the PEM fuel cell was generated by reducing the fuel cell temperature, and the accumulation of liquid water could block the gas path to the catalyst layer, leading to the degradation of the PEM fuel cell voltage, as can be seen in Figure 2a. Meanwhile, membrane dehydration was achieved by injecting non-humidified reactants to the PEM fuel cell to increase the temperature inside the stack and enable membrane dehydration to be developed, causing fuel cell performance degradation—depicted in Figure 2b. It should be noted that in Figure 3b, the initial voltage jump before 300 s is due to unstable PEM fuel cell performance at the starting period, after which the performance can be observed as being at a stable voltage. Based on previous studies [3,8,10,33], fuel cell flooding or membrane dehydration can cause fast fuel cell performance degradation, with the degradation rate being about 0.39 v/h and 0.25 v/h, respectively—thus, a test of only about 30 min was performed to obtain the test data for fuel cell flooding and membrane degradation, as this period was clearly able to cause the fuel cell voltage to drop.

From Figure 2, it is clear that both fuel cell flooding and membrane dehydration can lead to fuel cell performance decay—however, their degradation paths are different, which can be observed by the different voltage shapes in the transition period of these two faults. Moreover, it is also clear that performance due to fuel cell flooding can be effectively recovered by using proper mitigation strategies, while degradation due to membrane dehydration is irreversible and cannot be recovered. Therefore, it is necessary to distinguish these different failure modes, and appropriate control strategies need to be taken to extend PEM fuel cell performance.

### 3.2. Diagnosis Using Sensor Set with Different Sizes

In this section, measurements from three different sensor sets were employed to the PEM fuel cell fault diagnosis, including the voltage sensor only, all available sensors, and selected optimal sensors, using the techniques described in Section 2.3.

**Figure 4:**
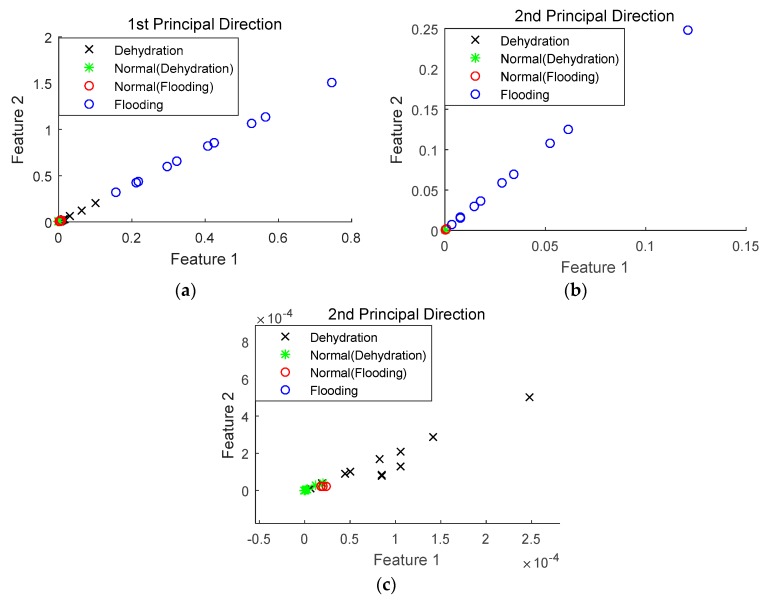
Fault diagnosis performance using selected sensors. (**a**) 1st principal direction; (**b**) 2nd principal direction; (**c**) zoom in the 2nd principal direction.

As described in Section 2.1, when only fuel cell voltage was used for fault diagnosis, wavelet packet transform was used to extract and generate normalized energies. The two highest energies were then selected for state discrimination, the diagnostic results of which are depicted in Figure 4.

Looking at the voltage information shown in Figure 3, the normal and faulty PEM fuel cell states can be clearly distinguished. However, the flooding and dehydration faults cannot be separated, indicating that various PEM fuel cell faults cannot be classified using the fuel cell voltage only, since both faults caused the voltage to drop at similar levels in the test.

Using the sensor selection technique described in Section 2.3, optimal sensors can be determined and their performance in fuel cell fault diagnosis can also be investigated. Compared to the diagnostic techniques used for the fuel cell voltage data, KPCA, described in Section 2.2, was employed to reduce the dimension of the dataset from optimal sensors, while wavelet packet transform was still applied to extract the features for classification. It should be mentioned that the high-dimensional dataset was projected in the first four principal directions, based on Equation (7). Figure 4 depicts the diagnostic results of the first two principal directions; the 3rd and 4th principal directions also show similar results, though not as clearly as the first two.

It can be seen from Figure 4 that with the inclusion of more sensors in the diagnosis, both normal and faulty states can be quite clearly distinguished. Moreover, the flooding and dehydration states can also be separated, indicating that it is possible to classify different PEM fuel cell failure modes with selected optimal sensors.

**Figure 5:**
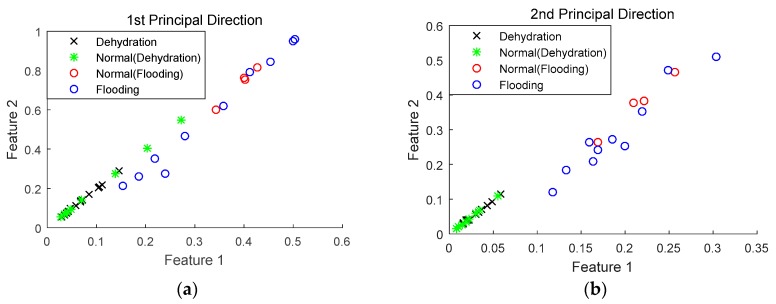
Fault diagnosis performance using all available sensors. (**a**) 1st principal direction; (**b**) 2nd principal direction.

The performance of fuel cell fault diagnosis using further increased sensors (all available sensors in this study) was also investigated. Figure 5 shows the diagnostic performance using all available sensors at the first two principal directions. It should be mentioned that the diagnostic techniques applied to all available sensors were the same as those used in the selected optimal sensors.

From Figure 5, it is clear that with a further increase of sensors in PEM fuel cell fault diagnosis, the diagnostic performance decreases significantly, even though fuel cell normal and faulty states cannot be distinguished. One possible reason for this is that several sensors used in the analysis are not sensitive to the PEM fuel cell performance variation, and will be more easily affected by measurement/environment noise—hence, the inclusion of these sensors will make a negative contribution to the PEM fuel cell fault diagnosis. It should be noted that the above results are comparable with those of previous studies [19,33], where the performance of all available sensors and selected sensors in identifying fuel cell flooding was investigated and results also demonstrate that the selected sensors can provide better diagnostic performance.

It can be concluded that with PEM fuel cell voltage information, the normal and faulty fuel cell states can be clearly classified, but reliable fault isolation cannot be provided as the voltage alone cannot provide enough information about degradation path variation due to different failure modes. With selected optimal sensors, more information can be provided, and fuel cell failure modes can be clearly distinguished—however, with a further increase in the sensor set, especially the inclusion of sensors which are not sensitive to changes in fuel cell performance, fuel cell diagnostic performance will reduce significantly, indicating the necessity of selecting proper sensors for PEM fuel cell fault diagnosis.

## 4. Conclusions

In this study, a comparative study was performed to investigate the effects of sensor set size on PEM fuel cell fault diagnosis. Wavelet packet transform was applied to sensor measurements to extract wavelet coefficients, from which generalized energies were calculated, and the two largest energies were used to classify different PEM fuel cell states. Moreover, in order to deal with the measurements from multiple sensors (selected optimal sensors and all available sensors in this study), KPCA was utilized to reduce the high-dimensional data set while retaining the useful information of the original data set.

Test data from the PEM fuel cell system was used to investigate the diagnostic performance using sensor sets of different sizes, including the voltage sensor, selected optimal sensors, and all available sensors. Three different fuel cell states were tested with the test bench, including the normal state, fuel cell flooding, and membrane dehydration. Results demonstrate that although PEM fuel cell faults can be detected correctly by using fuel cell voltage, fuel cell flooding and membrane dehydration cannot be distinguished. This is due to the fact that fuel cell voltage only cannot provide enough information to represent performance decay paths, due to various fuel cell faults (fuel cell flooding and membrane dehydration, in this study). However, with selected optimal sensors, PEM fuel cell faults can be detected and fuel cell flooding and membrane dehydration can also be isolated with good quality. With a further increase in sensor set size, i.e., including all available sensors in the analysis, fuel cell faults cannot be detected and various fuel cell faults also cannot be distinguished, indicating that the inclusion of sensors not sensitive to fuel cell performance change will contribute negatively to the fault diagnosis. Therefore, it can be summarized that in practical applications, the size of the sensor set for fuel cell fault diagnosis should be carefully determined in order to ensure reliable diagnostic performance with affordable computational effort.

## Figures and Tables

**Table 1:** Technical details of the PEM fuel cell system.

Parameter	Value
Membrane thickness (μm)	25
Active area (${\mathrm{cm}}^{2}$)	100
Platinum loading ($\mathrm{mg}/{\mathrm{cm}}^{2}$)	0.2
Gas diffusion thickness (μm)	415

**Table 2:** Sensors used in the fuel cell test.

No.	Sensor
1	Cathode outlet flow meter
2	Anode outlet flow meter
3	Cathode outlet humidification sensor
4	Anode outlet humidification sensor
5	Cathode outlet pressure gauge
6	Anode outlet pressure gauge
7	Stack thermocouple
8	Cathode outlet thermocouple
9	Anode outlet thermocouple
